# Prostate cancer in a family with a germline ATM pathogenic variant: a case report

**DOI:** 10.1186/s13053-026-00352-z

**Published:** 2026-08-12

**Authors:** Catherine Murphy, Justin Lorentz, Angelina Tryon, Danny Vesprini, Andrea Eisen, Steven A. Narod

**Affiliations:** 1 Odette Cancer Centre, Sunnybrook Health Sciences Centre, University of Toronto, 2075 Bayview Avenue, Toronto, ON Canada; 2 Women’s College Hospital, Toronto, ON Canada

**Keywords:** ATM, Prostate cancer, Hereditary cancer predisposition, Germline pathogenic variant, Familial cancer risk

## Abstract

**Introduction:**

Germline pathogenic variants (GPVs) in the in ataxia-telangiectasia mutated (*ATM*) gene are present in approximately 0.35% of the population, and are associated with increased risks of breast, pancreatic and prostate cancer.

**Case:**

We describe a family of Portuguese descent with a germline protein-truncating variant in *ATM (*c.450_453del) with a striking aggregation of prostate cancer and breast cancer on both the paternal and maternal lineages.

**Discussion:**

The family described here illustrates high penetrance for prostate and breast cancer and illuminates possible heterogeneity in the familial risk of prostate cancer according to the specific variant in the *ATM* gene.

## Background

In 1995, researchers identified the *ATM* gene and its association with ataxia–telangiectasia (AT) AT is a rare autosomal recessive disorder marked by cerebellar degeneration, oculocutaneous telangiectasia, and immune deficiency. The ATM protein encodes a PI3K-related serine/threonine protein kinase (PIKK) that plays a central role in the repair of DNA double strand breaks (DSBs) [1, 2]. Although the exact mechanism remains unclear [3–5] *ATM* GPVs predispose to several cancer sites by causing chromosomal instability due to defects in DNA repair that impair genomic integrity. Accurate cancer risk estimates for individuals carrying *ATM* GPVs have yet to be determined. The lifetime risk of breast cancer in heterozygous *ATM* GPV carriers was estimated to be 20–40% [6] and the lifetime risk of pancreatic cancer is estimated to be 5–10% [6]. Furthermore there is evidence for a 3-fold risk of gastric and a modestly elevated risk of colorectal cancer (1.5-fold increase) [7].

Evidence for elevated risk of prostate cancer risk with *ATM* is emerging, and several clinical studies suggest that *ATM* is associated with an aggressive form of prostate cancer. According to *The Cancer Genome Atlas*, approximately 7% of primary prostate tumor samples exhibit somatic or germline alterations in *ATM* [8, 13]. In 2024, Mukhtar et al. demonstrated that *ATM* protein-truncating variants (PTVs) are associated with a moderate increase in prostate cancer risk [9]. In a study by Carter et al. men with germline pathogenic variants in *BRCA2*,* BRCA1*, or *ATM* on active surveillance for prostate cancer were shown to have significantly higher rates of grade reclassification on repeat biopsies [10]. These data suggest that *ATM* variants contribute to prostate cancer susceptibility, in particular to aggressive disease [9]. This finding suggests that germline mutation status should be considered when counseling patients about the appropriateness of active surveillance.

## Case presentation

**Fig. 1:**
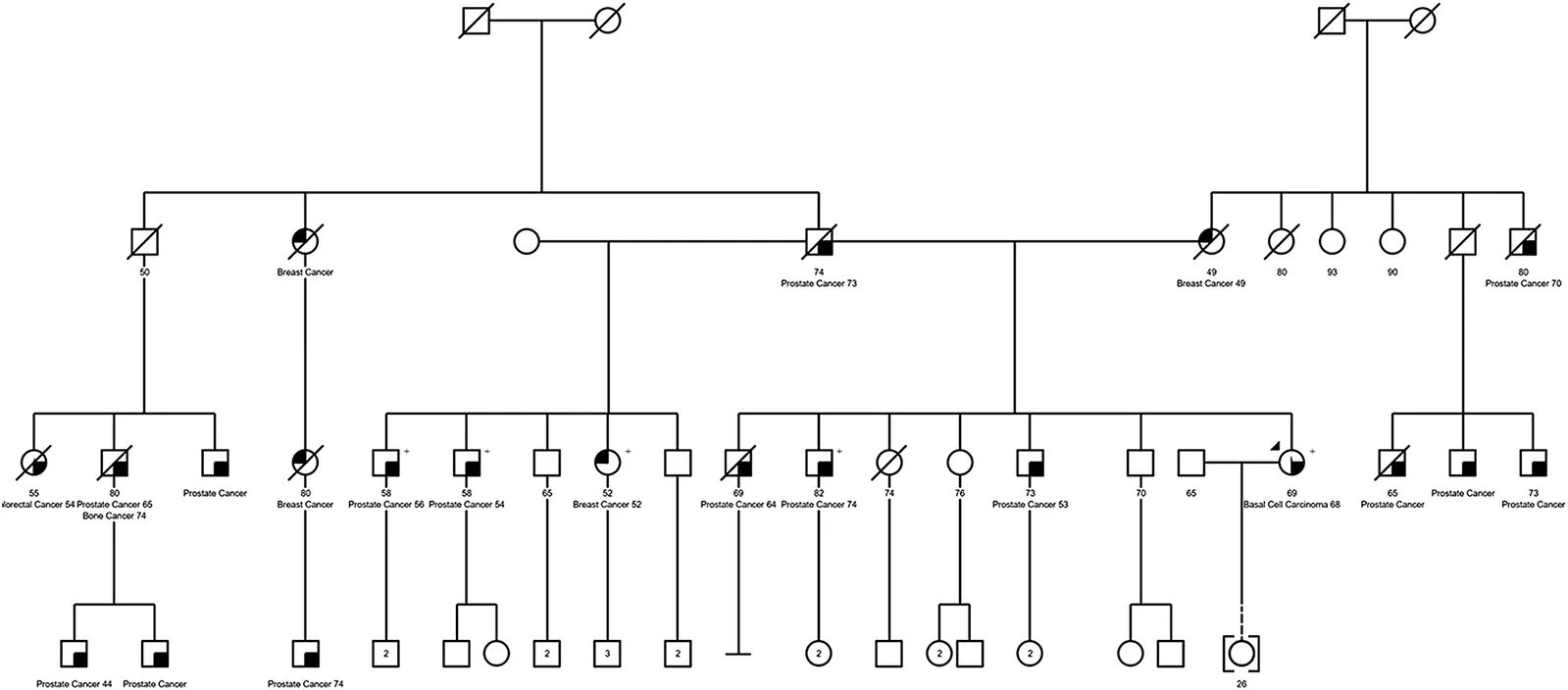
Pedigree of a family with the germline pathogenic variant *ATM* c.450_453del. The proband (arrow) is a female heterozygous carrier who presented with basal cell carcinoma. Multiple male relatives were affected by prostate cancer (shaded squares), and several female relatives developed breast cancer (shaded circles). Confirmed mutation carriers are indicated by central dots. Ages at diagnosis are shown below symbols where known. The pattern is consistent with autosomal-dominant transmission of *ATM*-associated cancer susceptibility through the paternal lineage

This case involves a family of Portuguese ethnicity. The family history revealed a striking aggregation of prostate cancer in both the paternal and maternal lineages (Fig. 1). The proband is a 68-year-old woman with a history of basal cell carcinoma who had a 19 gene panel preformed. The 19-gene breast/ovarian/prostate panel was offered based on her unexplained maternal history of cancer and not based on her BCC diagnosis. Testing confirmed that she carries the ATM c.450_453del variant.

### Paternal lineage

The proband’s father was diagnosed with prostate cancer at age 73 (*ATM* status unknown). Among the proband’s brothers, three of four full brothers and two of four paternal half-brothers developed prostate cancer, with ages at diagnosis ranging from 44 to 73 years. Pathology reports were available for two of her three affected brothers and both demonstrated intermediate risk Gealson 7 (3 + 4) disease, both of these brothers had radical prostatectomies and subsequently required salvage radiotherapy due to rising PSA post operatively.

Two paternal male cousins were also affected. Three of the cases were confirmed to be carriers of the ATM variant. Breast cancer was diagnosed in one paternal half-sister at age 52, one paternal female cousin at age 50, and a paternal aunt in her 70s. The *ATM* mutation appears to come from paternal lineage with one full brother, two paternal half-brothers and one paternal half-sister known to carry the mutation.

### Maternal lineage

Although no pathogenic variant has been identified on the maternal side, the family history also shows a significant cluster of prostate cancer, including the proband’s maternal uncle (diagnosed in his 70s) and three maternal male cousins (diagnosed between ages 65 and 73). The proband’s mother had breast cancer at age 49. No relatives on the maternal side had undergone germline testing at the time of reporting and there was no known history of consanguinity.

There are no known cases of pancreatic, ovarian, or hematologic malignancy reported. The pedigree (Fig. 1) demonstrates multigenerational clustering of prostate and breast cancers, consistent with an autosomal-dominant inheritance pattern of cancer susceptibility due to a germline *ATM* mutation. In addition, there is also an interesting cluster of prostate cancer coming from the maternal lineage with no known genetic mutation identified to date.

### Variant details

The germline pathogenic variant identified in this family is *ATM* c.450_453del (p.Leu150_Ser151insTer). This variant results in a frameshift and premature stop codon, predicted to produce a truncated, non-functional ATM protein [11]. Loss-of-function variants in *ATM* are known to be pathogenic [12, 13]. This variant is rare in population databases though has been previously reported in individuals with hereditary cancer phenotypes including reported predisposition to prostate cancer and is described as pathogenic by ClinVar [18–25].

## Discussion

In this family of Portuguese decent, the *ATM* germline frameshift pathogenic variant c.450_453del was associated with a remarkable familial clustering of cancer affecting both males and females, most prominently prostate cancer in males. GPVs in *ATM* are considered to be moderate penetrance prostate cancer risk genes [14]. The extent of cancer clustering in this family supports the view that *ATM* represents a pleiotropic cancer susceptibility gene. Possible explanations include allele-specific effects, genetic modifiers, or shared environmental exposures. Family history is a significant predictor of prostate cancer risk. The Prostate Cancer data Base Sweden (PCBaSe) demonstrated this relationship by examining 51,897 men who had brothers diagnosed with prostate cancer. By age 65, men with an affected brother had a 14.9% risk of developing prostate cancer compared with 4.8% in those without a family history. By age 75, the risks rose to 30.3% versus 12.9%, respectively, underscoring the strong familial influence on prostate cancer susceptibility [15]. Furthermore, a large prospective study of 203,000 twin pairs from Nordic countries (including 80,000 monozygotic and 123,000 same-sex dizygotic pairs) found a concordance rate of 38% among monozygotic twins and 22% among dizygotic twins. These findings suggest that up to 57% of prostate cancer risk may be attributable to heritable factors [16].

Together, these studies highlight that family history remains one of the strongest indicators of individual prostate cancer risk. While a substantial proportion of prostate cancer risk appears to be heritable at the population level, only a minority of cases are explained by variants in hereditary cancer predisposition genes, such as pathogenic variants in the ATM gene. This suggests that the clustering of prostate cancer observed in this family may reflect a combination of shared genetic susceptibility beyond a single variant together with potential shared environmental factors.

*ATM*-related practice guidelines from the American College of Medical Genetics and Genomics highlighted not just elevated lifetime risk for prostate cancer in *ATM* carriers but also increased risk for disease aggressiveness [4]. Mondschein et al. reported that germline *ATM* and *CHEK2* pathogenic variants were associated with an intermediate- to high-risk prostate cancer phenotype, supporting the role of these genes in aggressive disease biology. Similarly, within this family, both *ATM* variant carriers developed disease requiring treatment escalation, with salvage radiotherapy administered within one year of radical prostatectomy despite an initial Gleason score of 7 [17]. Rare variants associated with higher risk breast cancer have been reported largely in individuals of Northern and Western European descent [4, 5].

We must consider that prostate cancer is a polygenic disease, with individual risk influenced by a combination of effects from multiple genetic variants. Genome-wide association studies (GWAS) have identified over 100 loci associated with prostate cancer susceptibility. Together it is estimated that these variants account for 33% of familial prostate cancer [18–24].

Previous reports have described variability in cancer risk among *ATM* GPV carriers, with certain truncating or missense variants behaving as higher-penetrance alleles, particularly when present in families with extensive cancer aggregation. The American College of Medical Genetics and Genomics recognizes that certain missense variants, confer higher cancer risks and recommends tailored management for carriers of these alleles [25]. Our findings align with this emerging evidence, highlighting the heterogeneous nature of *ATM*-related cancer risk.

Clinically, this case underscores the importance of individualized risk assessment and surveillance strategies for carriers, even of variants typically considered moderate-risk, and supports consideration of *ATM* testing and intensified screening protocols in families with multiple related cancer diagnoses.

## Conclusion

This case demonstrates a striking aggregation of prostate and breast cancers within a family known to carry a pathogenic *ATM* GPV. Acknowledging this association supports the inclusion of *ATM* in hereditary cancer testing panels and highlights the clinical importance of early detection, risk-based surveillance, and cascade testing among at-risk family members. The clustering of prostate cancer on the maternal side in the absence of a pathogenic cancer predisposition gene highlights the importance of family history in determining a man’s risk of developing prostate cancer.

## Data Availability

No datasets were generated or analysed during the current study.
